# Association of sense of coherence, job stress and professional quality of life in emergency nurses: a multicenter cross-sectional study

**DOI:** 10.3389/fpsyg.2026.1811366

**Published:** 2026-07-10

**Authors:** Yue Lv, Jing Liu, Ying Sun, Chunmei Liu, Xinxia Li, Nuo Zhao

**Affiliations:** 1 Third Department of Health Care, The Second Medical Center and National Clinical Research Center for Geriatric Diseases of Chinese PLA General Hospital, Beijing, China; 2 Agency for Offices Administration of Central Military Commission, Beijing, China; 3 Department of Emergency, The Third Medical Center of Chinese PLA General Hospital, Beijing, China; 4 Nursing Department, The Third Medical Center of Chinese PLA General Hospital, Beijing, China

**Keywords:** emergency nurse, job stress, mediating effects, professional quality of life, sense of coherence

## Abstract

**Background:**

It is global issue that emergency nurses exhibit a high turnover rate due to excessive job stress and impaired professional quality of life (ProQOL). While sense of coherence (SOC), as a salutogenic psychological resource, may mediate these associations, its mediating role requires further exploration.

**Objective:**

To examine job stress, SOC, and ProQOL levels among Chinese emergency nurses and investigate the mediating effect of SOC between job stress and ProQOL.

**Methods:**

From May to August 2024, a total of 280 emergency nurses from four tertiary hospitals in Beijing, China were surveyed using a demographic questionnaire, the Chinese Nurse Job Stressors Questionnaire, the Professional Quality of Life Scale, and the Sense of Coherence Scale. Pearson correlation analyses, and structural equation modeling were conducted to examine the mediating role of SOC in the relationship between job stress and ProQOL.

**Results:**

Job stress was significantly associated with SOC and all three dimensions of ProQOL (*p* < 0.01). SOC partially mediated the associations between job stress and Compassion Satisfaction [indirect effect = −0.147, 95% CI (−0.214, −0.095)], Burnout [indirect effect = 0.089, 95% CI (0.042, 0.146)], and Compassion fatigue [indirect effect = 0.098, 95% CI (0.046, 0.158)].

**Conclusions:**

This study indicates that SOC partially mediated the relationship between job stress and ProQOL. Enhancing SOC may be beneficial for improving the job satisfaction and well-being of emergency nurses.

## 1 Introduction

Emergency departments represent a critical frontline healthcare environment characterized by unpredictability, frequent overcrowding, traumatic events and life-threatening conditions, and time-sensitive decisions-making on a daily basis (Adriaenssens et al., 2015; Salvarani et al., 2019; Valdez, 2019). In such settings, emergency nurses work under significantly higher job stress than those in other general departments, manifested through heavy workloads, high physical demands, and critical clinical situations (Bardhan et al., 2019; Jiaru et al., 2023). Studies have shown that serious turnover rates and human resources shortage of emergency staff have become a global issue (Valdez, 2019). Improving the well-being of emergency nurses has therefore received increasing attention.

Professional quality of life (ProQOL) is a multidimensional concept that comprehensively measures the healthcare professionals' overall job satisfaction and work experience (Joinson, 1992). The classic ProQOL framework proposed by Stamm comprises three key dimensions: (1) Compassion satisfaction (CS), refers to the fulfillment, motivation and personal accomplishment from effective caregiving (Abdul Rahman et al., 2017), (2) compassion fatigue (CF), a common phenomenon that individuals experience physical and mental exhaustion from chronic exposure to compassion-driven work-related stress (Stamm, 2005), and (3) burnout (BO) defined as a state of emotional exhaustion, depersonalization, and reduced personal accomplishment arising from work resource-demand imbalances (Yu and Gui, 2022).

Previous studies have indicated job stress is a core determinant of ProQOL, which has a direct impact on nurses' physical and mental health, work engagement, care quality and turnover rate (Abdul Rahman et al., 2017; Mohammadi et al., 2017; Mansouri et al., 2025; Ni et al., 2023; Remegio et al., 2021; Ruth-Sahd and Grim, 2021). According to stress-coping model Lazarus and Folkman's (1984), strain outcomes arise not from the stressor itself but from the dynamic interaction between the individual and the environment, mediated by cognitive appraisal and available coping resources. Therefore, in this study, job stress is conceptualized as emergency nurses' perceived exposure to stressors (e.g. workload and time allocation).

We propose that such stressor exposure may directly affect ProQOL, but also indirectly through individual cognition and psychological resources.

Sense of coherence (SOC) refers to a stable orientation reflecting an individual's tendency to perceive life as comprehensible, manageable and meaningful, which is an important psychological protective factor to cope with internal and external stimuli (Antonovsky, 1987; Machado et al., 2017). It consists of three components: Comprehensibility (perceiving stressors are structured, predictable, and explicable); Manageability (confidence in accessing resources to meet demands); Meaningfulness (motivation to engage with challenges as worthwhile investments; Antonovsky, 1987). Individuals with higher SOC are better able to conduct cognitive appraisal, apply flexible coping strategies, and mediate the impact of stress on their emotions (anxiety, burnout, and depression), and enhance psychological resilience. Previous studies have demonstrated the relationship between job stress and SOC (Danioni et al., 2025; Malinauskiene et al., 2009), as well as the relationship between SOC and ProQOL (Wilczek-Rużyczka et al., 2019a; Zerach and Levin, 2018; Zhang et al., 2023). Thus, we hypothesize that SOC may be a salutogenic resource that mediates the effects of job stress on ProQOL and constructs a structural equation model to examine the mediating role of SOC between job stress and ProQOL among emergency nurses.

**Figure 1:**
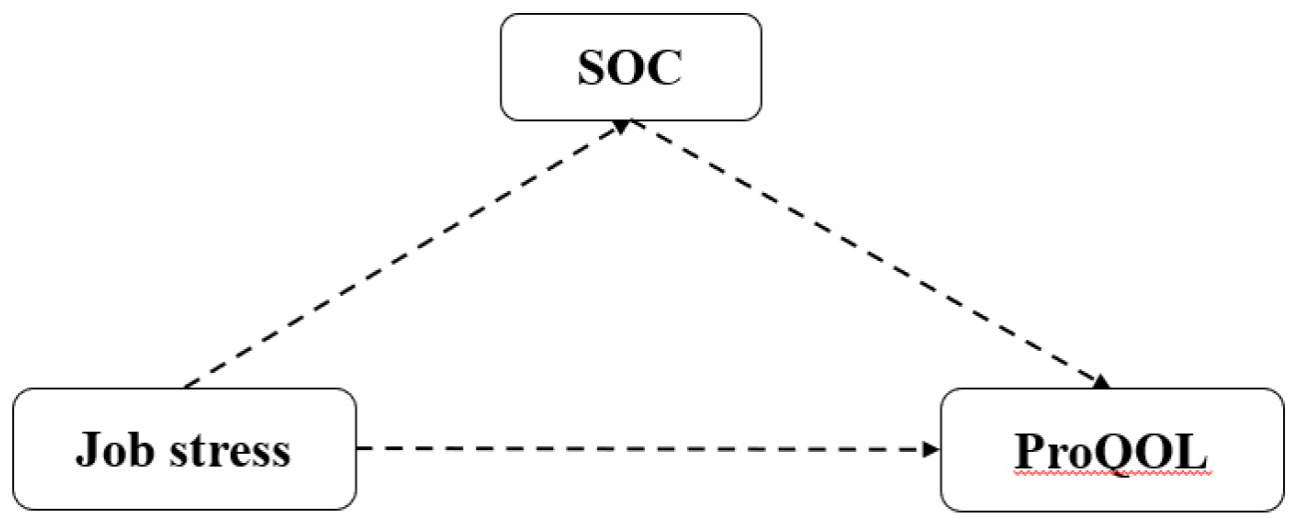
The theoretical model of this study.

Based on the extant evidence, we designed the research hypothesis model as shown in Figure 1 and put forward the following hypotheses:

*Hypothesis 1* Job stress is associated with SOC and ProQOL.
*Hypothesis 2* SOC directly affects ProQOL.
*Hypothesis 3* SOC mediates the relationship between job stress and ProQOL.

## 2 Methods

### 2.1 Study design

A descriptive, cross-sectional study.

### 2.2 Participants

The sample size was calculated using G\^*^Power 3.1 (Faul et al., 2007). Assuming a mediation model with three predictors, we used the parameters from prior literature (Kim and, 2011): *f*^2^ = 0.15 (medium), α = 0.05, and power = 0.80. The required minimum sample size was estimated to be 77. Considering the possibility of 20% invalid responses, the required sample size was 92. Ultimately, a total of 280 emergency nurses were conveniently recruited from four tertiary hospitals in Beijing, China, between May to August 2024. To be eligible, participants had to be (1) registered nurses, (2) have at least one year of frontline emergency experience, and (3) voluntary to join the study. We excluded those in managerial positions and nurses on sick leave, probation, or part-time contracts.

### 2.3 Measurements

#### 2.3.1 Demographic and work-related characteristics

A questionnaire was designed to collect the demographic and work-related characteristics of the participants, including gender, age, marital status, fertility status, professional rank, monthly income, and night shift frequency.

#### 2.3.2 Job stress

Job stress was assessed using the validated Chinese Nurse Job Stressors scale developed by Li (2000), which referred to Gray-Toft and Anderson (1981) and Wheeler and and Riding (1994) instruments and adapted for Chinese contexts. The 35-items scale covers 5 dimensions: Career development and salary benefit (7 items); Workload and time allocation (5 items); Working environment and resources (3 items); Patient care (11 items); Management and interpersonal relationship (9 items). Responses used Likert 4-point scale (1= strongly disagree, 4= strongly agree), with higher scores indicating greater perceived occupational stressors. The Cronbach's α coefficient of the scale in this study was 0.970.

#### 2.3.3 Sense of coherence (SOC)

Sense of Coherence Scale-13 (SOS-13) with 13 items developed by Antonovsky (1993) and translated to Chinese version by Bao and Liu (2005) was used for measurement. The scale consisted of 3 dimensions of comprehensibility (5 items), manageability (4 items) and meaningfulness (4 items). Likert 7-point scoring was used for each item. Eight items were positively scored, and the remaining five were scored in reverse. Higher total scores (range: 13–91) indicated a stronger sense of coherence and levels were categorized as low (13–63), moderate (64–79), or high (80–91) based on previous study (Xue et al., 2025). The Cronbach's α coefficient in this study was 0.940.

#### 2.3.4 Professional quality of life (ProQOL)

Professional quality of life was measured using the Chinese version of Stamm's (2005) Professional Quality of Life scale, which was translated and validated by Xing et al. (2013). The scale consists of three dimensions: compassion satisfaction (CS), burnout (BO) and compassion fatigue (CF)—each measured by 10 items. Items are rated on a Likert 5-point ranging from 1 (never) to 5 (very often). Five items (1, 4, 15, 17, and 29) are reverse scored. Each subscale score ranges from 10 to 50, with higher scores indicating higher levels of CS, BO, or CF. According to established cutoffs (Hui et al., 2024), scores ≤ 22, 23–41, and ≥42 correspond to low, moderate and high levels, respectively. The Cronbach's coefficients of each subscale were 0.897, 0.923, and 0.914, respectively.

### 2.4 Data collection

Data collection utilized a 15-min online survey on an anonymous online website platform named WenJuanXing. We approached the head nurses of the selected departments and explained the survey purpose to eligible nurses. To ensure the scientific validity of the research and the authenticity of the data, the entire investigation was conducted anonymously. All items in the questionnaire had been set as mandatory to eliminate the bias caused by incomplete answers. Additionally, the WenJuanXing platform restricted allowed only one submission per IP address to prevent duplicate responses.

### 2.5 Ethical considerations

Before conducting the survey, we consulted the Ethics Committee of the Chinese PLA General Hospital and received the reply that formal ethical review was not required for this research as we collected the experiences of emergency nurses through an anonymous online survey. Ethical principles were strictly adhered throughout the research to protect participants' rights in accordance with the Declaration of Helsinki and China's ethical regulations (China., N.H.C.O., 2023). The purpose of this survey was written on the first page of the questionnaire for respondents. After consulting the head nurses of the selected departments, we approached the eligible nurses and explained the survey purpose to them. Participants were assured that they could decline or withdraw from this study at any time before submission without any impact on their work. All collected data were strictly confidential, used solely for this research, and reported only in aggregate form. All participants gave informed e-consent to participate in the study.

### 2.6 Statistical analysis

IBM SPSS version 27.0 and AMOS 26.0 software were used for statistical analysis. First, Harmans' single-factor test was conducted to assess common method bias (Podsakoff et al., 2003). Continuous variables were described using mean ± standard deviation (SD) if the dataset met the criteria for a normal distribution, and the categorical data were described as counts and percentages. Then independent samples *t*-test (for two-group variables) and Kruskal-Wallis *H* test (for variables with three or more groups) were utilized to examine the differences in CS, BO, and CF scores across sociodemographic subgroups. Subsequently, Pearson correlation analysis was used to explore the interrelationship among the primary variables given that the data were approximately normal. Finally, we used AMOS 26.0 to conduct structural equation modeling (SEM) to investigate the mediating effect of SOC in the relationship between job stress and ProQOL while controlling for sociodemographic characteristics. Maximum likelihood estimation was used to estimate the path coefficients of the model. Indirect effects were tested using bootstrapping (2000 resamples) with bias corrected 95% confidence intervals (CIs). A two tailed *P* < 0.05 was considered statistically significant.

## 3 Results

### 3.1 Sample characteristics

A total of 280 eligible nurses completed and submitted the online survey. After data quality checks, no invalid responses were identified; therefore, all 280 cases were included in the final analysis. The samples characteristics are presented in Table 1.

**Table 1:** Characteristics of the included emergency nurses (*n* = 280).

Characteristics		N (%)	CS		BO		CF	
		Mean ±SD	t/F (*P*)	Mean ±SD	t/F (*P*)	Mean ±SD	t/F (*P*)
Gender	Male	23 (8.2)	32.7 ± 9.4		30.2 ± 9.5		28.8 ± 8.9	
	female	257 (91.8)	32.1± 8.9	0.3 (0.750) ^a^	29.6 ± 7.9	0.3 (0.736)^a^	28.0 ± 7.3	0.5 (0.617)^a^
Age(years)	< 30	143 (51.1)	31.7 ± 9.0		30.1 ± 7.9		28.6 ± 7.3	
	30–40	110 (39.3)	32.0 ± 9.3		29.3 ± 8.6		27.6 ± 7.8	
	>40	27 (9.6)	34.7 ± 6.6	1.8 (0.406)^b^	28.6 ± 6.7	0.8 (0.677)^b^	27.2 ± 7.4	1.2 (0.558)^b^
Marital status	unmarried	133 (47.5)	32.8 ± 8.4		29.5 ± 7.7		27.6 ± 6.9	
	Married	147 (52.5)	31.5 ± 9.5	1.2 (0.227) ^a^	29.9 ± 8.3	−0.4 (0.680)^a^	28.5 ± 7.8	−0.9 (0.359)^a^
Numbers of children	0	152 (54.3)	32.3 ± 8.6		29.9 ± 7.6		28.0 ± 7.0	
	1	75 (26.8)	32.1 ± 9.7		29.1 ± 8.5		28.0 ± 8.0	
	≥2	53 (18.9)	31.8 ± 9.2	0.0 (0.991)^b^	29.9 ± 8.7	0.3 (0.871)^b^	28.3 ± 7.7	0.2 (0.923)^b^
Professional title	Junior nurse	60 (21.4)	31.9 ± 8.5		29.6 ± 7.9		28.1 ± 6.6	
	Staff nurse	125 (44.6)	31.7 ± 8.9		30.3 ± 7.7		28.7 ± 7.7	
	Charge nurse	95 (33.9)	32.8 ± 9.4	0.8 (0.687) ^b^	28.9 ± 8.5	0.8 (0.672) ^b^	27.3 ± 7.5	1.6 (0.456)^b^
Average number of night shifts per month (times)	0	48 (17.1)	33.7 ± 8.3		30.2 ± 7.4		28.7 ± 5.8	
	1–4	34 (12.1)	32.3 ± 10.9		27.7 ± 8.5		26.9 ± 7.9	
	5–8	81 (28.9)	30.7 ± 7.9		30.7 ± 7.9		29.0 ± 7.8	
	9–12	63 (22.5)	32.1 ± 8.9		29.1 ± 7.9		27.9 ± 7.9	
	≥13	54 (19.3)	32.8 ± 9.7	4.3 (0.366)^b^	29.5 ± 8.6	2.9 (0.573)^b^	27.2 ± 7.2	2.8 (0.600)^b^
Average monthly income(¥)	≤ 5000	13 (4.6)	34.0 ± 8.9		26.8 ± 8.5		26.4 ± 7.9	2.7 (0.617)^b^
	5001–8000	130 (46.4)	31.6 ± 9.2		29.4 ± 8.2		28.2 ± 7.7	
	8001–10000	92 (32.9)	32.9 ± 8.4		29.6 ± 6.9		27.7 ± 6.9	
	10000–15000	37 (13.2)	31.5 ± 9.2		31.9 ± 9.2		29.8 ± 7.8	
	≥15001	8 (2.9)	31.6 ± 10.9	1.8 (0.765)^b^	29.8 ± 10.4	2.6 (0.630)^b^	26.1 ± 6.1	

a - t test, b - Kruskal-Wallis H test.

The majority were female (91.8%) and under 30 years old (51.1%, *n* = 143). *T*-test and Kruskal-Wallis H tests showed no significant associations between any demographic variable and CS, BO, and CF scores (all *p* > 0.05).

### 3.2 Common method bias

Given that all variables were self-reported at a single point, we assessed the risk of common-method bias using Harman's single-factor test. An unrotated exploratory factor analysis on all items from the three scales showed that the first factor accounted for 34.1% of the total variance, below the 40% threshold (Podsakoff et al., 2003), indicating that common-method bias is not a major concern.

### 3.3 Descriptive statistical analysis of the study variables

As shown in Table 2, the average score of job stress was 109.87 ± 22.54. The SOC score was 60.27 ± 14.60, with 55.9% at a moderate level. CS, BO, and CF scores were (32.12 ± 8.98), (29.68 ± 8.03), and (28.08 ± 7.42), respectively. 201 participants (71.8%) had moderate levels of CS, and 38 participants (13.6%) reported high levels of CS. The majority reported moderate levels of BO (*n* = 205, 73.2%) and CF (*n* = 202, 72.1%).

**Table 2:** Scores of nurses' work stress, SOC and ProQOL (*n* = 280).

Variables (item numbers)	Total scores (Mean ±SD)	Low *N* (%)	Moderate *N* (%)	High *N* (%)
*Job stress*	109.87 ± 22.54			
Career development and salary benefits (7)	21.36 ± 4.08			
Workload and time allocation (5)	15.11 ± 4.03			
Working environment and resources (3)	9.80 ± 2.34			
Patient care (11)	35.46 ± 8.12			
Management and interpersonal relationship (9)	28.14 ± 6.80			
*SOC*	60.27 ± 14.60	104 (37.2)	156 (55.7)	20 (7.1)
Comprehensibility (5)	21.65 ± 6.56			
Manageability (4)	18.96 ± 4.89			
Meaningfulness (4)	19.66 ± 4.84			
*Compassion satisfaction (10)*	32.12 ± 8.98	41 (14.6)	201 (71.8)	38 (13.6)
*Burnout (10)*	29.68 ± 8.03	53 (18.9)	205 (73.2)	22 (7.9)
*Compassion fatigue (10)*	28.08 ± 7.42	67 (23.9)	202 (72.1)	11 (3.9)

### 3.4 Correlation analysis of job stress, SOC, and ProQOL

Table 3 presents the Pearson correlation analysis results among the study variables. Job stress was significantly negatively correlated with SOC (*r* = −0.56) and CS (*r* = −0.29), and significantly positively correlated with BO (*r* = 0.43) and CF (*r* = 0.44) (both *P* < 0.01). SOC was positively correlated with CS (*r* = 0.44) and negatively correlated with BO (*r* = −0.41) and CF (*r* = −0.41) (both *P* < 0.01).

**Table 3:** Correlation analysis of nurses' job stress, SOC and ProQOL (*n* = 280).

Variables	1	2	3	4	5	6	7	8
1 Job stress	1							
2 SOC	−0.56^**^	1						
3 Comprehensibility	−0.67^**^	0.86^**^	1					
4 Manageability	−0.41^**^	0.93^**^	0.65^**^	1				
5 Meaningfulness	−0.36^**^	0.91^**^	0.60^**^	0.92^**^	1			
6 Compassion satisfaction	−0.29^**^	0.44^**^	0.38^**^	0.41^**^	0.40^**^	1		
7 Burnout	0.43^**^	−0.41^**^	−0.43^**^	−0.36^**^	−0.30^**^	−0.51^**^	1	
8 Compassion fatigue	0.44^**^	−0.41^**^	−0.40^**^	−0.38^**^	−0.33^**^	−0.50^**^	0.82^**^	1

^**^ p < 0.01.

### 3.5 Mediating effect of SOC between job stress and ProQOL

We ran three separate mediation models using SEM in AMOS 26.0. In each model, job stress (independent variable) and SOC (mediator) were entered as latent variables, while CS, BO, CF (outcome variables) were entered as observed variables. Although Table 1 showed no significant associations between any demographic variable and CS, BO, and CF, we still included them as covariates in the model to adjust for their potential influence.

**Figure 2:**
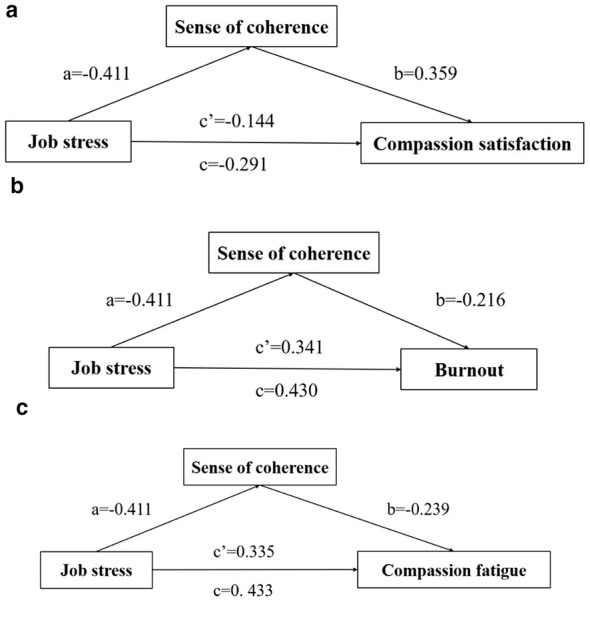
**(A)** Path diagram for model 1 (Outcome: Compassion satisfaction) with standardized coefficients. **(B)** Path diagram for model 2 (Outcome: Burnout) with standardized coefficients. **(C)** Path diagram for model 3 (Outcome: Compassion fatigue) with standardized coefficients. *Notes:* a, the effect of job stress on SOC; b, the effect of SOC on CS/BO/CF; c, the total effect of job stress on CS/BO/CF; c', the direct effect of job stress on CS/BO/CF.

Table 4 and Figure 2 summarized the results. Across all three models, job stress was negatively associated with SOC (β= −0.411, *P* < 0.001). SOC showed significant associations with CS (β = 0.359), BO (β = −0.216) and CF (β = −0.239) (all *P* < 0.001). The direct effects were also significant for all three outcomes (job stress → CS: β−0.144; job stress → BO: β = 0.341; job stress → CF: β = 0.335; all *P* < 0.05).

**Table 4:** Path coefficients for the three mediation models (*N* = 280).

Path	Standardized coefficient	Unstandardized coefficient	SE	*P*
Job stress → SOC	−0.411	−0.192	0.032	^***^
SOC → CS	0.359	1.071	0.203	^***^
Job stress → CS	−0.144	−0.201	0.084	0.017
SOC → BO	−0.216	−0.575	0.165	^***^
Job stress → BO	0.341	0.425	0.074	^***^
SOC → CF	−0.239	−0.59	0.153	^***^
Job stress → CF	0.335	0.386	0.068	^***^

SE = standard error; ^***^ p < 0.001.

As shown in Table 5, the indirect effects in three models were all significant [CS: −0.147, 95% CI (−0.214, −0.095); BO: 0.089, 95% CI (0.042, 0.146); CF:0.098, 95% CI (0.046,0.158)]. These results indicate that SOC partially mediates the association between nurses' job stress and CS, BO and CF, with the indirect effect accounted for 50.5% for CS, 28.1% for BO, and 29.1% for CF.

**Table 5:** Indirect effects and bootstrap confidence for the three mediation models.

Path	Effect	95% CI		Relative effect (%)
		Lower limit	Upper limit	
Job stress → SOC → CS	−0.147	−0.214	−0.095	50.5
Job stress → SOC → BO	0.089	0.042	0.146	28.1
Job stress → SOC → CF	0.098	0.046	0.158	29.1

CI, Confidence Interval; Relative effect, (indirect effect / total effect) × 100%.

## 4 Discussion

This study aimed to examine the relationship between job stress, SOC, and ProQOL, providing a comprehensive understanding of the internal mechanism and pathway through which perceived job stressors are associated with emergency nurses' ProQOL. To our knowledge, it is the first to evaluate the mediating role of SOC between job stress and ProQOL in emergency nurses.

In this study, the mean item score for job stress was 3.13 ± 0.64, which is slightly higher than that reported by Yu et al. (2.88 ± 0.60) in emergency nurses from Sichuan Province, China. Among the five domains, work environment and resources scored the highest, while workload and time allocation scored the lowest, suggesting that the participating hospitals may have relatively adequate staffing, while improving the work environmental conditions may be a priority for reducing job stress of emergency nurses.

As for ProQOL, the mean scores for CS, BO, and CF were 32.12 ± 8.98, 29.68 ± 8.03, and 28.08 ± 7.42, respectively, with 71.8, 73.2, and 72.1% of cases falling into the moderate groups. These findings are similar to prior studies conducted in emergency nurses (Hunsaker et al., 2015), indicating that a notable proportion of emergency nurses experience moderate levels of compassion satisfaction, burnout and compassion fatigue. Interestingly, no significant associations were found between any sociodemographic characteristics and the three ProQOL dimensions, which contrast with some previous studies (Hunsaker et al., 2015), but aligns with study O'Callaghan et al.'s (2020) and a meta-analysis in oncology nurses (Algamdi, 2022). One possible explanation is in the high-stress emergency setting, workplace contextual stressors may outweigh individual characteristics in influencing ProQOL, emergency nurses are facing changeable situations, and their CS, BO, and CF might change on a daily basis (Eddy et al., 2017). Consistent with this, recent studies have increasingly focused on environmental and organizational contributors (e.g., workload, workplace violence, and leadership; Cosentino et al., 2023; Itzhaki et al., 2018) and psychological resilience factors (e.g., emotional intelligence, social support; Francis-Wenger, 2024; Musio et al., 2025; Yu and Gui, 2022) as key determinants of ProQOL. Therefore, understanding the pathway between job stress and ProQOL and developing targeted strategies is imperative.

As predicted, the current research suggested that SOC partially mediate the associations between job stress and CS, BO, and CF, consistent with previous studies conducted in other nurse populations (Wilczek-Rużyczka et al., 2019b; Yanfeng, 2021). According to Antonovsky (1987), individuals with high SOC are more likely to adopt adaptive strategies to cope with stressful situations, such as positive cognitive appraisal and seeking available resources. Yanfeng (2021) noted that under the same stress level, nurses with high SOC level tend to view stress as a meaningful growth and challenge, gain achievement from managing it, and believe that stress can be controlled through effective use of resources. Consequently, they report higher CS and lower BO and CF. Thus, strengthening SOC may be an effective approach to reducing the negative impacts of excessive job stress on nurses' well-being.

Notably, the mean score of SOC was 60.27 ± 14.60, with 55.9% at a moderate level, similar to previous studies (Furong and Hui, 2022; Yanfeng, 2021). Among the three dimensions, Meaningfulness scored the highest (4.92 ± 1.21), while Comprehensibility scored the lowest (4.33 ± 1.31). This reflects that emergency nurses gain a strong sense of value from life-saving work, but the unpredictable and uncontrollable work environment keeps them in a state of high sensitivity and perceived disorder. Existing research has shown that SOC is a dynamic psychological resource and can be strengthened by targeted training (Kretowicz and Bieniaszewski, 2015). A multilevel framework is recommended: at the organizational level, addressing root causes (e.g., workload, role ambiguity) and implementing tailored solutions (e.g., flexible scheduling, institutional commitments) can enhance comprehensibility and manageability (Masanotti et al., 2020); at the individual level, cognitive-behavioral interventions (e.g., online cognitive behavioral therapy) can help vulnerable nurses modify negative thinking patterns and rebuild career identification (Pollock et al., 2020).

This study has several limitations. First, the cross-sectional design capture data at a single point and limits the ability to infer causal relationships between the variables. Longitudinal studies are recommended to examine how these associations evolve over time. Second, the present study collected data from 4 tertiary hospitals in Beijing, China, the generalizability of our findings may be limited to similar populations.

## 5 Conclusions

Overall, this study found that job stress was associated with emergency nurses' professional quality of life, and that SOC may partially mediate this association. Future research and practice should develop targeted strategies to enhance nurses' SOC from both individual and organizational perspectives, so as to help nurses better cope with high levels of stress in the emergency department, achieve better professional quality of life, and thereby provide high-quality care and maintain career stability.

## Data Availability

The original contributions presented in the study are included in the article/supplementary material, further inquiries can be directed to the corresponding author.
